# Supplementary material for: Multimorbidity Patterns among People with Type 2 Diabetes Mellitus: Findings from Lima, Peru
Source: Int J Environ Res Public Health. 2022 Jul 30;19(15):9333. doi: 10.3390/ijerph19159333 (PMC9367906; doi:10.3390/ijerph19159333)
Supplement: Supplementary file 1 [file ijerph-19-09333-s001.zip › ijerph-1815535-supplementary.pdf]

Multimorbidity Patterns among People with Type 2 Diabetes Mellitus: Findings from Lima, Peru

Supplemental Material

Table S1. Definitions of morbidities used in the analyses.

Morbidity	Definition
Anemia	D50, D51, D52, D53, D54
Anxiety	F40, F41
Arthropathy	M05, M06, M07, M08, M09, M10, M11, M12, M13, M14, M15, M16, M17, M18, M19
Atrial fibrillation	I48
Cancer	C00 to C97
Cerebrovascular disease	I60, I61, I62, I63, I64
Chronic back pain	M54
Chronic kidney disease	N18
COPD	J44
Dementia/Alzheimer	F00, G30
Depression	F32, F33
Dyslipidemia	E78<br>Total cholesterol ≥200 mg/dL, LDL≥100 mg/dL, HDL<40 mg/dL, or triglycerides≥150 mg/dL
Ischemic heart disease	I20, I21, I22, I23, I24, I25
Hypertension	I10, I11<br>Systolic blood pressure≥140mmHg, diastolic blood pressure≥90 mmHg
Hypothyroidism	E02, E03
Heart failure	I50
Obesity	E66<br>Body mass index≥30 kg/m^2
Tuberculosis	A15, A16, A17, A18, A19
Urinary lithiasis	N20, N21, N22, N23

COPD = Chronic obstructive pulmonary disease.

Table S2. Distribution of morbidities according to the number of chronic conditions (n = 5243).

	Number of chronic conditions			
**Morbidity**	**1**	**2**	**3+**	**p-value**
	**(n = 3145)**	**(n = 933)**	**(n = 384)**	
Obesity	1506 (47.9%)	933 (69.1%)	384 (85.5%)	<0.001
Hypertension	786 (25.0%)	692 (51.2%)	318 (70.8%)	<0.001
Dyslipidemia	372 (11.8%)	432 (32.0%)	280 (62.4%)	<0.001
Hypothyroidism	179 (5.7%)	261 (19.3%)	176 (34.2%)	<0.001
Arthropathy	96 (3.1%)	143 (10.6%)	106 (23.6%)	<0.001
Chronic kidney disease	44 (1.4%)	83 (6.1%)	64 (14.3%)	<0.001
Anemia	64 (2.0%)	63 (4.7%)	45 (10.0%)	<0.001
Chronic back pain	53 (1.7%)	54 (4.0%)	53 (11.8%)	<0.001
Anxiety	45 (1.4%)	41 (3.0%)	25 (5.6%)	<0.001
Cerebrovascular disease	32 (1.0%)	11 (0.8%)	3 (0.7%)	0.51
Tuberculosis	12 (0.4%)	4 (0.3%)	1 (0.2%)	<0.001
Cancer	28 (0.9%)	6 (0.4%)	0 (0.0%)	0.11
Heart ischemic disease	24 (0.8%)	9 (0.7%)	4 (0.9%)	0.43
Atrial fibrillation	27 (0.9%)	4 (0.3%)	3 (0.7%)	0.02
Heart failure	15 (0.5%)	10 (0.7%)	5 (1.1%)	0.13
Urinary lithiasis	11 (0.4%)	8 (0.6%)	4 (0.9%)	0.26
Depression	8 (0.3%)	3 (0.2%)	1 (0.2%)	0.31
COPD	2 (0.1%)	2 (0.2%)	0 (0.0%)	0.74
Dementia	2 (0.1%)	1 (0.1%)	0 (0.0%)	0.72

COPD = Chronic obstructive pulmonary disease
